# Genotype–phenotype correlation of biallelic DUOX2 mutations in transient and permanent congenital hypothyroidism

**DOI:** 10.1210/jendso/bvag081

**Published:** 2026-04-13

**Authors:** Sheng-Bin Liang, Chih-Ya Cheng, Yun-Ru Chen, Yann-Jang Chen, Chia-Feng Yang, Yung-Hsiu Lu, Dau-Ming Niu

**Affiliations:** Department of Pediatrics, Taipei Veterans General Hospital, Taipei 11217, Taiwan; Department of Pediatrics, Taipei Veterans General Hospital, Taipei 11217, Taiwan; Department of Pediatrics, Taipei Veterans General Hospital, Taipei 11217, Taiwan; Department of Pediatrics, Taipei Veterans General Hospital, Taipei 11217, Taiwan; Department of Life Sciences and Institute of Genome Sciences, National Yang Ming Chiao Tung University, Taipei 112304, Taiwan; Department of Pediatrics, Taipei Veterans General Hospital, Taipei 11217, Taiwan; School of Medicine, National Yang Ming Chiao Tung University, Taipei 112304, Taiwan; Department of Pediatrics, Taipei Veterans General Hospital, Taipei 11217, Taiwan; Department of Pediatrics, Taipei Veterans General Hospital, Taipei 11217, Taiwan; Institute of Clinical Medicine, School of Medicine, National Yang Ming Chiao Tung University, Taipei 112304, Taiwan; Institute of Molecular and Genomic Medicine, National Health Research Institutes, Miaoli 35053, Taiwan

**Keywords:** congenital hypothyroidism, thyroid dyshormonogenesis, DUOX2 mutations, transient congenital hypothyroidism, genotype–phenotype correlation, treatment outcome

## Abstract

**Context:**

Congenital hypothyroidism (CH) due to DUOX2 mutations shows marked clinical heterogeneity. While some patients recover thyroid function during early childhood, others require long-term levothyroxine (LT4) therapy. The determinants of this variability remain unclear.

**Objective:**

To determine whether baseline biochemical severity, thyroid morphology, bone maturation, and biallelic DUOX2 variant severity are associated with thyroid function recovery in DUOX2-related CH.

**Methods:**

We retrospectively studied 71 patients with biallelic pathogenic DUOX2 variants, classified into 3 outcome groups: LT4 discontinued by age 4 years (*n* = 19), LT4 discontinued after age 4 years (*n* = 25), and continued LT4 with biochemical worsening requiring dose increase (*n* = 27). Baseline thyroid function tests, thyroid volume, and bone maturation were compared among groups. Bone maturation was assessed by distal femoral epiphyseal area (DFEA) and categorized as normal (DFEA ≥0.22 cm^2^) or delayed. Genotype–phenotype associations were examined, including variability among patients with identical biallelic genotypes.

**Results:**

Baseline thyroid function did not differ across groups (TSH *P* = .222; T3 *P* = .314; T4 *P* = .422; free T4 *P* = .505; thyroglobulin *P* = .363). Thyroid volume (*P* = .246) and bone maturation (*P* = .238) were not significantly different. The distribution of putative severe DUOX2 alleles did not differ (*P* = .862). Identical genotypes were observed across different outcome groups, indicating divergent clinical courses.

**Conclusion:**

Baseline biochemical severity, thyroid volume, bone maturation, and DUOX2 variant severity did not reliably predict recovery in DUOX2-related CH. Substantial within-genotype heterogeneity suggests contributions from compensatory H_2_O_2_-generating pathways and/or other modifying factors.

Congenital hypothyroidism (CH) is the most common neonatal endocrine disorder, with a global incidence of approximately 1 in 2000-4000 live births [1-3]. Early detection through newborn screening and timely initiation of levothyroxine (LT4) are essential to prevent irreversible neurodevelopmental impairment. Etiologically, CH is classically categorized into thyroid dysgenesis (TD) and thyroid dyshormonogenesis (TDH). TD reflects abnormal thyroid development—agenesis, hypoplasia, ectopy, or hemiagenesis—and accounts for the majority of permanent CH cases (approximately 80-85%). In contrast, TDH accounts for the remaining 15% to 20% and results from inherited defects in thyroid hormone biosynthesis, secretion, or action, with pathogenic variants described across nearly all steps of the thyroid hormone–producing pathway [3-5].

Taiwan is an iodine-sufficient region [6-8]. The reported prevalence of moderate-to-severe permanent CH is approximately 1 in 5788 live births [9], with about 20% attributable to dyshormonogenesis; among these, mutations in TPO and TG represent a large proportion [10, 11]. Beyond classic permanent CH, mild forms—including cases that appear severe in the neonatal period but evolve into milder dysfunction—and neonatal transient hypothyroidism that resolves by 2-3 years of age are increasingly recognized in routine practice. In Taiwan, the estimated incidence of transient or mild CH may be as high as 1 in 1000 live births [12], underscoring the growing clinical need to distinguish children who will recover endogenous thyroid function from those who will require prolonged therapy.

Recent studies have identified DUOX2 variants as a leading genetic cause of mild and transient CH, particularly in East Asian populations [13, 14]. DUOX2 encodes a transmembrane flavoprotein at the apical membrane of thyroid follicular cells that generates hydrogen peroxide (H_2_O_2_), an essential cofactor for thyroid peroxidase (TPO) during iodination and coupling reactions in thyroid hormone synthesis. DUOX2 activity depends on the maturation factor DUOXA2, and pathogenic variants in either gene can impair hormonogenesis [15, 16]. Importantly, DUOX2-related CH exhibits striking clinical heterogeneity, ranging from transient neonatal hypothyroidism to permanent CH requiring long-term LT4. Even among patients with biallelic DUOX2 variants, thyroid function may normalize within early childhood in some individuals, while others remain LT4-dependent. A few recent studies have suggested that genotype–phenotype correlation is inconsistent in biallelic DUOX2-related CH and may not reliably predict long-term outcome [17-19]. This heterogeneity complicates decisions about LT4 duration, requiring a balance between avoiding premature withdrawal during vulnerable neurodevelopmental windows and preventing unnecessary prolonged therapy. Therefore, we investigated whether routinely available, biologically relevant clinical measures can predict long-term LT4 requirement (transient vs persistent disease) in children with biallelic DUOX2-related CH. We evaluated parameters reflecting biochemical severity (screening and confirmatory thyroid function tests), thyroid structure and potential functional reserve or tissue activity (thyroid volume and serum thyroglobulin), and integrated early-life thyroid hormone exposure (bone maturation). Using what is, to our knowledge, the largest single-center cohort systematically followed from newborn screening through long-term outcomes with standardized longitudinal reassessment, we evaluated long-term thyroid outcomes. We further examined within-genotype variability by comparing patients with identical biallelic genotypes but divergent clinical courses. This approach clarified the utility and limitations of baseline predictors and highlighted the potential role of modifying factors in thyroid function recovery.

## Methods

### Study design and participants

This retrospective single-center cohort study was conducted at a tertiary referral center in Taiwan. Infants were referred after abnormal results from the nationwide congenital hypothyroidism (CH) newborn screening program. We included 71 patients who underwent genetic testing and were found to carry biallelic DUOX2 variants, with longitudinal follow-up sufficient to ascertain treatment outcomes. The study was approved by the institutional review board of Taipei Veterans General Hospital (IRB No. 2025-07-031BC).

### Newborn screening and confirmatory evaluation

In Taiwan, newborn screening is conducted through a nationwide, government-supported program. For the entire NBS panel (including congenital hypothyroidism), heel-prick dried blood spot (DBS) samples are routinely collected between 48 and 72 hours of life after at least 24 hours of feeding. DBS cards are air-dried and dispatched on the same day to a central screening laboratory, and results are typically available ∼3 days after sample receipt. For infants flagged with abnormal DBS TSH, follow-up is guided by a national referral algorithm, while the exact work-up may be individualized by the receiving center and clinical context. In general, DBS TSH 10 to μU/mL prompts repeat DBS testing (usually coordinated by the birth hospital); 20 to 40 μU/mL prompts referral for confirmatory venous TFTs (serum TSH and free T4, ± T3/T4), with additional evaluations (eg, thyroid autoantibodies, bone age, and/or thyroid ultrasonography) performed as clinically indicated; and >40 μU/mL prompts urgent referral for confirmatory venous TFTs and a more comprehensive evaluation, including additional studies and thyroid imaging as indicated (including Tc-99m thyroid scintigraphy when DBS TSH is >80 μU/mL). Because this stepwise system reflects real-world clinical prioritization, the timing of the confirmatory work-up varies by initial screening TSH level and clinical urgency; however, confirmatory venous TFTs and related evaluations, as indicated, are typically obtained within the first 1 to 3 weeks of life.

### Genetic evaluation and case selection

Since 2017, families have been offered a partially subsidized CH-targeted next-generation sequencing panel (CH-NGS), which includes DUOX2, DUOXA2, GLIS3, IGSF1, IYD, SLC26A4, SLC5A5, TG, THRA, THRB, TPO, and TSHR. Beginning in 2020, partially subsidized whole-exome sequencing (WES) or whole-genome sequencing (WGS) has also been available for broader genetic evaluation. Enrollment in genetic testing was not based on CH severity; rather, inclusion depended on parental consent and partial out-of-pocket payment in our setting.

### Clinical assessments

We extracted demographic data, newborn screening DBS TSH, confirmatory venous thyroid function tests (TFTs), and longitudinal outcomes from medical records. Baseline laboratory evaluation at confirmatory diagnosis included serum TSH, free T4, total T4, total T3, and thyroglobulin (TG). Given the marked dispersion of TSH in DUOX2-related CH, TSH values are summarized as mean ± SD and median (min–max, range), and between-group differences in baseline TFTs were assessed using the Kruskal-Wallis test. During part of the study period, the laboratory reported TG values above the assay upper reporting limit as “>300 ng/mL” without dilution. For continuous analyses, these censored values were excluded. To retain information, TG was additionally analyzed as a prespecified categorical variable (≥300 vs <300 ng/mL), and group distributions were compared using the Fisher–Freeman–Halton exact test.

Thyroid volume was assessed by ultrasonography when available. Total thyroid volume (mL) was calculated as the sum of both lobes using the rotational ellipsoid method (lobe volume = length × width × depth × 0.479), based on the Brunn/WHO approach [20, 21].

Bone maturation at diagnosis was assessed using the distal femoral epiphyseal area (DFEA) measured on frontal knee radiographs. DFEA was quantified by digital planimetry (manual outlining of the distal femoral epiphysis on a digital workstation), and the larger DFEA from the 2 knees was used for analysis. Because DFEA values were widely distributed and knee radiographs were obtained at variable postnatal ages in routine practice, bone maturation was analyzed both continuously (DFEA) and categorically (normal vs delayed; DFEA ≥0.22 cm^2^), using a previously published cutoff [22]. This approach improves interpretability and mitigates sensitivity to floor effects and timing-related variability. To minimize confounding by prematurity, only infants with gestational age >38 weeks were included in the bone maturation analysis.

### LT4 withdrawal protocol, outcome definitions, and group classification

Patients with eutopic thyroid glands were considered for LT4 discontinuation between ages 2 and 3 years if LT4 dosing had remained stable without escalation and clinical status was appropriate. LT4 was tapered stepwise, reducing the dose by approximately one-third every 2 to 3 months, guided by serial TFTs, until complete cessation. After LT4 discontinuation, thyroid function was reassessed at 1 month and then every 3 months for 12 months. CH was classified as transient only if euthyroidism was maintained throughout this 12-month period, defined as TSH <6 mIU/L with both free T4 and total T4 maintained in the age-appropriate upper-normal range. Given that DUOX2-related CH may exhibit TSH fluctuation over time, ongoing surveillance was continued thereafter, typically every 6-12 months, with closer monitoring during periods of increased thyroid hormone demand (eg, puberty and pregnancy). Patients were classified into 3 outcome groups based on treatment course: Group 1, LT4 discontinued by age 4 years (*n* = 19); Group 2, LT4 discontinued after age 4 years (*n* = 25); and Group 3, LT4 continued beyond age 4 years at the time of analysis (*n* = 27). Not all Group 3 patients underwent a complete withdrawal attempt: some had persistently elevated TSH (>8 mIU/L) on stable LT4 without tapering, requiring LT4 dose escalation, whereas others showed TSH >10 mIU/L during tapering before complete cessation. In these cases, LT4 was maintained, and complete withdrawal was considered clinically inappropriate. Individual-level clinical and TFT data for all patients across the 3 groups are provided in Tables S1–S3 [23], including post-withdrawal TFTs and, for Group 3, reassessment TFT results.

## Results

We compared baseline thyroid function, perinatal characteristics, thyroid morphology, bone maturation, and LT4 dosing across 3 outcome groups: Group 1 (LT4 discontinued by age ≤4 years, *n* = 19), Group 2 (LT4 discontinued after age >4 years, *n* = 25), and Group 3 (LT4 continued beyond age 4 years at the time of analysis, *n* = 27). Baseline characteristics are summarized by group in Table 1, with reassessment/withdrawal data summarized in Table 2. Individual-level baseline clinical and genotypic data are provided in Tables S1–S3 (including Tables S1A–S3A) [23], and individual TFT results during withdrawal/reassessment are provided in Table S4 [23].

**Table 1:** Baseline characteristics of patients with biallelic DUOX2-related CH by LT4 outcome

Parameter	Discontinued ≤ 4 yrs (*N* = 19)	Discontinued > 4 yrs (*N* = 25)	Not Discontinued (*N* = 27)	*P*-value
1st NBS TSH (μIU/mL) mean ± SD, median (range)	39.9 ± 34.8 33.8 (14.7-54.6)	37.1 ± 35.3 24.2 (16.0-40.3)	67.2 ± 64.9 41.0 (20.4-98.9)	.162
Initial confirmatory visit				
Initial confirmatory visit age (day)	14.5 ± 6.3	14.3 ± 7.5	17.6 ± 15.9	.927
TSH (μIU/mL) mean ± SD median (range)	120.9 ± 93.1 82.0 (38.3-357.6)	102.8 ± 65.4 75.0 (19.5-247.7)	145.8 ± 106.8 123.8 (22.1-446.6)	.222
T3 (ng/dL)	147.2 ± 67.8	123.3 ± 49.4	122.5 ± 34.6	.314
T4 (µg/dL)	4.8 ± 2.0	4.4 ± 2.6	4.3 ± 2.1	.422
Free T4 (ng/dL)	0.67 ± 0.20	0.67 ± 0.30	0.63 ± 0.33	.505
TG (ng/mL)	1838.1 ± 1073.1 1772.0 (250-3321)	1326.0 ± 681.1 1778.0 (397-1841)	1464.0 ± 1339.3 1147.5 (141-5502)	.363
Thyroid volume (mL)	2.79 ± 2.03	2.01 ± 1.23	1.80 ± 0.93	.246
Gestational age (weeks)	38.4 ± 1.1	38.5 ± 1.8	37.9 ± 1.4	.094
Birth body weight (g)	3175.5 ± 475.2	3056.7 ± 414.8	3044.3 ± 395.4	.580
DFEA (cm^2^)	0.23 ± 0.10	0.21 ± 0.12	0.18 ± 0.13	.238
Initial dose (µg/day)	35.3 ± 10.0	33.1 ± 7.4	38.6 ± 11.6	.150
Initial dose/weight	6.8 ± 2.5	6.2 ± 1.9	7.4 ± 3.7	.359
Discontinuation age (years)	3.2 ± 0.7	7.2 ± 2.4	NA	—

Abbreviations: DBS, dried blood spot; DFEA, distal femoral epiphyseal area; free T4, free thyroxine; LT4, levothyroxine; NA, not available; NBS, newborn screening; T3, total triiodothyronine; T4, total thyroxine; TG, thyroglobulin; TSH, thyroid-stimulating hormone.

Data are presented as mean ± SD unless otherwise indicated. For DBS TSH and serum TSH, data are additionally shown as median (range). Groups were defined by LT4 course: discontinued ≤4 years, discontinued >4 years, and continued beyond 4 years at last follow-up. *P* values were calculated using the Kruskal–Wallis test for continuous variables, as appropriate. TG values reported as “>300” (assay upper reporting limit without dilution) were not used for continuous-variable summary statistics.

**Table 2:** Thyroid hormone profiles during LT4 tapering/withdrawal or reassessment by outcome group

Parameter	Discontinued ≤ 4 years (*N* = 19)	Discontinued > 4 years (*N* = 25)	Not Discontinued (*N* = 27)	*P*
TSH (µIU/mL)	2.85 ± 1.4	3.14 ± 0.7	10.90 ± 3.7	<.001
T3 (ng/dL)	147.4 ± 25.6	139.9 ± 25.3	129.3 ± 29.7	.127
T4 (µg/dL)	8.26 ± 1.1	7.78 ± 1.2	8.28 ± 1.4	.413
Free T4 (ng/dL)	1.37 ± 0.3	1.44 ± 0.2	1.4 ± 0.2	.371

Abbreviations: free T4, free thyroxine; LT4, levothyroxine; T3, total triiodothyronine; T4, total thyroxine; TSH, thyroid-stimulating hormone.

For Group 3, measurements were obtained during biochemical worsening on routine LT4 therapy or during tapering when complete withdrawal was considered clinically inappropriate. Data are presented as mean ± SD. *P* values were calculated using the Kruskal–Wallis test.

### Baseline thyroid function and birth characteristics

First DBS TSH and baseline thyroid function at confirmatory diagnosis did not differ across groups (first DBS TSH *P* = .162; confirmatory TSH *P* = .222; T3 *P* = .314; total T4 *P* = .422; free T4 *P* = .505). Given the wide dispersion of several laboratory measures, particularly TSH, results are presented as mean ± SD and median (range) (Table 1). Birth characteristics, including gestational age and birth weight, were also similar across groups (Table 1). Individual-level values are provided in Tables S1–S3 [23].

### Bone maturation and LT4 dose at initiation

Bone maturation, assessed by distal femoral epiphyseal area (DFEA), did not differ among groups (*P* = .238; Table 1). When dichotomized using the predefined cutoff (DFEA ≥0.22 cm^2^), the proportion of infants classified as having normal epiphyseal development also did not differ significantly across groups (*P* = .188; Table 3). Initial LT4 dosing, expressed as absolute and weight-adjusted doses, was comparable among groups (*P* = .150 and *P* = .359, respectively; Table 1).

**Table 3:** Distribution of bone maturation categories (DFEA) across LT4 outcome groups

Group	DFEA ≥ 0.22 cm^2^	DFEA < 0.22 cm^2^	Total (*N*)
Discontinued ≤ 4 years	9 (52.9%)	8 (47.1%)	17
Discontinued > 4years	9 (47.4%)	10 (52.6%)	19
Not discontinued	4 (23.5%)	13 (76.5%)	17
Total	22	31	53

Values are *n* (%).DFEA, distal femoral epiphyseal area. Bone maturation was categorized using the predefined cutoff (DFEA ≥0.22 cm^2^) [22]. Analysis includes infants with available DFEA measurements (gestational age >38 weeks). Fisher–Freeman–Halton exact test (two-sided), *P* = 0.188.

### Thyroid volume and thyroglobulin

Thyroid volume and serum TG were evaluated as markers of thyroid morphology and tissue activity. Neither thyroid volume nor TG differed among the 3 outcome groups (*P* = .246 and *P* = .363, respectively; Table 1). Consistent results were observed when TG was analyzed categorically (≥300 vs <300 ng/mL; Fisher-Freeman-Halton exact test, *P* = .093; Table S5) [23]. Individual-level thyroid volume and TG data are provided in Tables S1–S3 [23].

### Thyroid function at LT4 reassessment/withdrawal

Thyroid function parameters obtained during LT4 tapering, withdrawal, or reassessment (TSH, free T4, total T4, and total T3) are summarized at the group level in Table 2, with detailed individual-level results provided in Table S4 [23]. These data include measurements obtained during attempted tapering/withdrawal, as well as episodes of biochemical worsening during routine LT4 treatment in Group 3, in whom complete withdrawal was considered clinically inappropriate.

### DUOX2 variant severity and within-genotype variability

To explore genotype–phenotype associations, we compared the distribution of putative “severe” DUOX2 alleles across outcome groups. Because comprehensive functional data are unavailable for most DUOX2 variants and residual activity cannot be reliably inferred for individual missense changes—some of which may be as deleterious as nonsense or frameshift variants—classification of variant severity is inherently limited. For exploratory analyses, we therefore applied a conventional assumption and defined presumed severe alleles as nonsense or frameshift variants, which are more likely to result in loss of function. Using this definition, the distribution of nonsense or frameshift alleles did not differ across the 3 outcome groups (*P* = .862), indicating that the presence or number of presumed severe alleles was not associated with LT4 discontinuation status (Table 4). We further examined within-genotype variability by identifying patients with identical biallelic DUOX2 genotypes across different outcome groups (Table 5). Several identical genotypes were observed in patients with divergent clinical courses; for example, c.1588A > T (p.Lys530Ter)/c.3693 + 1G > T, c.2654G > T (p.Arg885Leu)/c.3693 + 1G > T, and c.1588A > T (p.Lys530Ter)/c.2654G > A (p.Arg885Gln) were present in both discontinuation and continued-treatment groups. These findings support substantial within-genotype heterogeneity and suggest that DUOX2 genotype severity alone does not fully explain thyroid function recovery.

**Table 4:** Distribution of presumed severe DUOX2 alleles by LT4 treatment outcome group

Study group	0 Severe allele	1 Severe allele	2 Severe alleles	Total (*N*)
Discontinued ≤ 4 years	9 (47.4%)	8 (42.1%)	2 (10.5%)	19
Discontinued > 4 years	10 (40.0%)	14 (56.0%)	1 (4.0%)	25
Not discontinued	11 (40.7%)	14 (51.9%)	2 (7.4%)	27
Total	30 (42.2%)	36 (52.1%)	5 (5.6%)	71

Values are *n* (%). Presumed severe alleles were defined as nonsense or frameshift variants. Fisher–Freeman–Halton exact test (two-sided), *P* = .862.

**Table 5:** Clinical features of patients with identical biallelic DUOX2 genotypes and divergent LT4 outcomes

Genotype (biallelic DUOX2 variants)	Group— case no.	TSH (µIU/mL)	T3 (ng/dL)	T4 (µg/dL)	fT4 (ng/dL)	DFEA (cm^2^)	BBW (g)	GA (weeks)	Initial LT4 Dose µg/day (µg/kg/day)
Allele1: c.1588A > T, p.Lys530Ter Allele2: c3693 + 1G > T, p.?	G1-04	135.9	173	7.1	0.70	0.07	4700	39	25.0 (4.63)
	G1-15	106	198	6.7	0.73	0.29	3330	39	50.0 (8.93)
	G3-11	72.4	168	8.4	1.13	*N*/A	3300	39	37.9 (7.15)
Allele1: c.2654G > T, p.Arg885Leu Allele2: c.3693 + 1G > T, p.?	G2-25*	*N*/A	*N*/A	*N*/A	*N*/A	*N*/A	3100	39	29.6 (5.19)
	G3-27*	110	*N*/A	*N*/A	1.00	*N*/A	2788	37	33.3 (8.12)
Allele1: c.1588A > T, p.Lys530Ter Allele2: c.2654G > A, p.Arg885Gln	G2-02	113.9	113	3.6	0.59	0.05	3020	38	42.7 (7.36)
	G3-06	85.3	162	3.4	0.43	0.42	2957	38	50.0 (11.90)
Allele1: c.3329G > A, p.Arg1110Gln Allele2: c.2654G > A, p. Arg885Gln	G1-01	357.6	82	3.2	0.50	0.09	3170	40	42.86 (7.79)
	G2-01	247.7	81	3.0	0.50	0.21	3020	39	33.3 (6.28)
	G2-19	175.0	79	1.6	0.40	0.29	2810	39	33.3 (5.41)

Abbreviations: BW, birth weight; DFEA, distal femoral epiphyseal area; fT4, free thyroxine; GA, gestational age; LT4, levothyroxine; NA, not available; T3, total triiodothyronine; T4, total thyroxine; TSH, thyroid-stimulating hormone.

Outcome groups were defined by LT4 course: G1, discontinued ≤4 years; G2, discontinued >4 years; G3, continued beyond 4 years at last follow-up. Case IDs are formatted as Gx-yy (group-case number). Identical genotypes appearing across groups represent different individuals. Cases G2-25 and G3-27 are sisters sharing the same biallelic genotype but assigned to different outcome groups. Initial LT4 dose is presented as absolute dose (µg/day) and weight-adjusted dose (µg/kg/day). * Initial LT4 treatment may have been initiated at referring hospitals for the cases marked with an asterisk.

## Discussion

Biallelic DUOX2 variants are increasingly recognized as an important cause of congenital hypothyroidism (CH), yet clinical presentation and long-term outcomes are highly heterogeneous. Patients may exhibit transient or persistent CH with wide variation in LT4 dose requirements and treatment duration [13, 16, 24-27]. In our cohort, we observed substantial phenotypic variability even among individuals sharing identical biallelic DUOX2 genotypes, underscoring the limited prognostic value of genotype alone.

We systematically assessed routinely available baseline parameters and genotype features in 71 patients with biallelic DUOX2 variants identified through newborn screening and followed longitudinally. Patients were stratified by treatment course into 3 outcome groups (LT4 discontinued by age ≤4 years, discontinued after age >4 years, and continued beyond age 4 years). Across these groups, first DBS TSH and confirmatory baseline thyroid function (TSH, T3, total T4, free T4) did not differ significantly, and perinatal characteristics were also similar. Markers reflecting thyroid structure and tissue activity—thyroid volume and serum thyroglobulin (TG)—were likewise comparable across groups and were not predictive of long-term LT4 requirement in this cohort. Collectively, these findings suggest that commonly available baseline clinical, biochemical, and structural measures do not reliably distinguish children who will recover thyroid function from those who will require prolonged therapy in biallelic DUOX2-related CH.

Bone maturation assessed by distal femoral epiphyseal area (DFEA) also did not significantly differ between outcome groups, whether analyzed as a continuous measure or dichotomized using a predefined cutoff. Although delayed bone maturation was frequent given the fact that FT4 levels are only just below the normal range, interpretation may consider the evolving biochemical course characteristic of DUOX2-related CH; a single confirmatory venous TFT obtained days to weeks after birth may not fully represent thyroid hormone exposure across late fetal life and early infancy, whereas skeletal maturation may reflect integrated exposure over a broader window. This may partly explain why bone maturation did not align closely with contemporaneous confirmatory TFT values.

Genotype-based severity stratification similarly showed limited predictive value. Using a conventional exploratory assumption, we defined presumed severe alleles as nonsense or frameshift variants; however, the distribution of these alleles did not differ across outcome groups. Moreover, multiple identical biallelic genotypes were observed in patients with divergent clinical trajectories, ranging from early LT4 discontinuation to ongoing therapy beyond age 4 years. These genotype-concordant but phenotype-discordant cases support substantial within-genotype heterogeneity and suggest that DUOX2 variant type alone does not fully explain thyroid function recovery.

A plausible explanation for this clinical heterogeneity lies in the functional redundancy and compensatory mechanisms within the thyroid hormone biosynthesis pathway. Both DUOX1 and DUOX2 enzymes generate hydrogen peroxide (H_2_O_2_), a critical substrate for thyroid hormone synthesis [28, 29]. However, DUOX1 is expressed at significantly lower levels—approximately one-fifth that of DUOX2 [30]. During the neonatal period, the physiological demand for thyroid hormone is 5-7 times higher than in adults [31, 32]. In this context, DUOX1-derived H_2_O_2_ alone may be inadequate, leading to hypothyroidism in individuals with DUOX2 deficiency. As the demand for thyroid hormone declines with age, DUOX1-mediated H_2_O_2_ production may become sufficient to sustain euthyroidism, thereby enabling recovery from CH despite persistent DUOX2 dysfunction [33].

The compensatory function of DUOX1 is considered a plausible primary mechanism for recovery in patients with DUOX2-related congenital hypothyroidism, as it may partially sustain the hydrogen peroxide (H_2_O_2_) supply necessary for thyroid hormone synthesis. Other potential H_2_O_2_-generating systems—such as NADPH oxidase 4 (NOX4), which has been reported in thyroid tissue—might also contribute, although their role in thyroid hormonogenesis remains uncertain [34, 35]. The complexity of clinical presentation in patients with DUOX2 mutations highlights the need to explore additional compensatory pathways and unidentified genetic or environmental modifiers that may influence disease expression.

From a clinical perspective, our findings reinforce the importance of standardized longitudinal reassessment and carefully monitored LT4 tapering/withdrawal trials rather than reliance on baseline markers. Notably, recent data indicate that recurrent hypothyroidism may occur even after prolonged drug-free intervals in DUOX2-related CH, including the need for retreatment after 6 to 8 months and even after approximately 10 years without therapy [19]. Therefore, although we classified patients as transient only after maintaining biochemical euthyroidism for ≥12 months post-withdrawal, long-term surveillance remains warranted.

### Limitations

This study has several limitations. First, interpretation of variant severity is constrained by the absence of functional assays for most DUOX2 variants. Residual enzymatic activity cannot be reliably inferred from sequence changes alone—particularly for missense variants, some of which may be as deleterious as truncating variants—so severity classification based solely on mutation type is inherently imprecise. Accordingly, our genotype analyses relied on a pragmatic exploratory approach focused on truncating (nonsense/frameshift) variants, and future studies incorporating in vitro functional assays or validated activity measures will be important to refine genotype–phenotype inference. Second, the timing of confirmatory testing and ancillary evaluations (eg, ultrasound-based thyroid volume, thyroglobulin, and knee radiographs for DFEA) varied in routine clinical practice, which may reduce sensitivity to detect subtle between-group differences. Third, although patients classified as transient maintained biochemical euthyroidism for ≥12 months after LT4 withdrawal, the duration of follow-up after discontinuation was not uniform and recurrent hypothyroidism has been reported after prolonged drug-free intervals; therefore, late relapse could have been missed in some individuals without extended surveillance. Finally, this was a single-center cohort, and while large for a rare disorder, the sample size limits statistical power for small effects and subgroup analyses; multicenter and prospective studies will be valuable to confirm these findings and better define predictors of long-term outcomes.

## Conclusion

In this single-center cohort with biallelic DUOX2 variants detected by newborn screening, baseline DBS TSH, confirmatory TFTs, thyroid volume, bone maturation, initial LT4 dose, and truncating variants did not distinguish children who discontinued LT4 from those requiring ongoing therapy. The finding that identical genotypes can follow markedly different clinical trajectories highlights the complexity of DUOX2-related CH. The potential compensatory role of DUOX1 and other as-yet-unidentified modifying factors warrants further investigation.

## Data Availability

The data supporting the findings of this study are included in the article and its supplemental materials. The supplemental tables have been deposited in Figshare (DOI: 10.6084/m9.figshare.31053529).

## References

[1] Delange F . Neonatal screening for congenital hypothyroidism in Europe. Report of the newborn committee of the European thyroid association. Acta Endocrinol Suppl (Copenh). 1979;223:3‐29. 285560

[2] Fisher DA, Dussault JH, Foley TP, Jr., et al Screening for congenital hypothyroidism: results of screening one million North American infants. J Pediatr. 1979;94(5):700‐705. 87512 10.1016/s0022-3476(79)80133-x

[3] Medeiros-Neto G, Knobel G, DeGroot LJ. Genetic disorders of the thyroid hormone system. In: Baxter JD, ed. Genetics in Endocrinology. Lippincott Williams & Wilkins; 2002:375‐402.

[4] Knobel M, Medeiros-Neto G. An outline of inherited disorders of the thyroid hormone generating system. Thyroid. 2003;13(8):771‐801. 14558921 10.1089/105072503768499671

[5] Refetoff S, Dumont J, Vassart G. Thyroid disorders. In: Valle DL, Antonarakis S, Ballabio A, Beaudet AL, Mitchell GA, eds. The Online Metabolic and Molecular Bases of Inherited Disease. 2019. Accessed April 9, 2025. https://ommbid.mhmedical.com/

[6] Wang FF, Tang KT, Pan WH, Won JG, Hsieh YT, Huang CJ. Iodine Status of Taiwanese population in 2013: 10 years after changing from mandatory to voluntary salt iodization. Food Nutr Bull. 2018;39(1):75‐85. 29117737 10.1177/0379572117738883

[7] Su GY, Yeh CC, Yang SJ, et al Assessment of iodine nutritional status and gestational thyroid function reference ranges during the first trimester of pregnancy in Taiwan. J Chin Med Assoc. 2024;87(6):590‐596. 38651854 10.1097/JCMA.0000000000001099 PMC12718866

[8] Huang CJ, Li JZ, Hwu CM, et al Iodine concentration in the breast milk and urine as biomarkers of iodine nutritional Status of lactating women and breastfed infants in Taiwan. Nutrients. 2023;15(19):4125. 37836409 10.3390/nu15194125 PMC10574722

[9] Tsai WY, Lee JS, Chao MC, et al Prevalence of permanent primary congenital hypothyroidism in Taiwan. J Formos Med Assoc. 1995;94(5):271‐273. 7613262

[10] Niu DM, Hwang B, Chu YK, Liao CJ, Wang PL, Lin CY. High prevalence of a novel mutation (2268 insT) of the thyroid peroxidase gene in Taiwanese patients with total iodide organification defect, and evidence for a founder effect. J Clin Endocrinol Metab. 2002;87(9):4208‐4212. 12213873 10.1210/jc.2002-020153

[11] Niu DM, Hsu JH, Chong KW, et al Six new mutations of the thyroglobulin gene discovered in Taiwanese children presenting with thyroid dyshormonogenesis. J Clin Endocrinol Metab. 2009;94(12):5045‐5052. 19837936 10.1210/jc.2009-0646

[12] Niu DM, Lin CY, Hwang B, Jap TS, Liao CJ, Wu JY. Contribution of genetic factors to neonatal transient hypothyroidism. Arch Dis Child Fetal Neonatal Ed. 2005;90(1):F69‐F72. 15613581 10.1136/adc.2003.039065 PMC1721821

[13] Maruo Y, Takahashi H, Soeda I, et al Transient congenital hypothyroidism caused by biallelic mutations of the dual oxidase 2 gene in Japanese patients detected by a neonatal screening program. J Clin Endocrinol Metab. 2008;93(11):4261‐4267. 18765513 10.1210/jc.2008-0856

[14] Narumi S, Muroya K, Asakura Y, Aachi M, Hasegawa T. Molecular basis of thyroid dyshormonogenesis: genetic screening in population-based Japanese patients. J Clin Endocrinol Metab. 2011;96(11):E1838‐E1842. 21900383 10.1210/jc.2011-1573

[15] Corvilain B, van Sande J, Laurent E, Dumont JE. The H_2_O_2_-generating system modulates protein iodination and the activity of the pentose phosphate pathway in dog thyroid. Endocrinology. 1991;128(2):779‐785. 1846588 10.1210/endo-128-2-779

[16] Maruo Y, Nagasaki K, Matsui K, et al Natural course of congenital hypothyroidism by dual oxidase 2 mutations from the neonatal period through puberty. Eur J Endocrinol. 2016;174(4):453‐463. 26742565 10.1530/EJE-15-0959

[17] Sun F, Wan JP, Zhang NN, et al Clinical outcomes of congenital hypothyroidism due to DUOX2 biallelic mutations after levothyroxine withdrawal. Thyroid. 2025;35(10):1120‐1128. 40916794 10.1177/10507256251372195

[18] Baz-Redón N, Antolín M, Clemente M, et al Patients with thyroid dyshormonogenesis and DUOX2 variants: molecular and clinical description and genotype-phenotype correlation. Int J Mol Sci. 2024;25(15):8473. 39126042 10.3390/ijms25158473 PMC11313534

[19] Uehara E, Abe K, Tanase-Nakao K, et al Molecular and clinical features of congenital hypothyroidism due to multiple DUOX2 variants. Thyroid. 2024;34(7):827‐836. 38757580 10.1089/thy.2024.0046

[20] Brunn J, Block U, Ruf G, Bos I, Kunze WP, Scriba PC. [Volumetric analysis of thyroid lobes by real-time ultrasound (author's transl)]. Dtsch Med Wochenschr. 1981;106(41):1338‐1340. Volumetrie der Schilddrüsenlappen mittels Real-time-Sonographie. 7274082 10.1055/s-2008-1070506

[21] Johnson A, Edwards C, Reddan T. A review of sonographic thyroid volume and iodine sufficiency in children: an Australian perspective. Australas J Ultrasound Med. 2020;23(1):33‐38. 34760580 10.1002/ajum.12189 PMC8411731

[22] Niu DM, Hwang B, Tiu CM, et al Contributions of bone maturation measurements to the differential diagnosis of neonatal transient hypothyroidism versus dyshormonogenetic congenital hypothyroidism. Acta Paediatr. 2004;93(10):1301‐1306. 15499948

[23] Liang S-B, Cheng C-Y, Chen Y-R, et al 2026. Data from: genotype–phenotype correlation of Biallelic DUOX2 mutations in transient and permanent congenital hypothyroidism. Figshare. doi:10.6084/m9.figshare.31053529. PMC13191362 42181674

[24] Moreno JC, Klootwijk W, van Toor H, et al Mutations in the iodotyrosine deiodinase gene and hypothyroidism. N Engl J Med. 2008;358(17):1811‐1818. 18434651 10.1056/NEJMoa0706819

[25] Cangul H, Aycan Z, Kendall M, et al A truncating DUOX2 mutation (R434X) causes severe congenital hypothyroidism. J Pediatr Endocrinol Metab. 2014;27(3-4):323‐327. 24127536 10.1515/jpem-2013-0314

[26] Jin HY, Heo SH, Kim YM, et al High frequency of DUOX2 mutations in transient or permanent congenital hypothyroidism with eutopic thyroid glands. Horm Res Paediatr. 2014;82(4):252‐260. 25248169 10.1159/000362235

[27] Muzza M, Rabbiosi S, Vigone MC, et al The clinical and molecular characterization of patients with dyshormonogenic congenital hypothyroidism reveals specific diagnostic clues for DUOX2 defects. J Clin Endocrinol Metab. 2014;99(3):E544‐E553. 24423310 10.1210/jc.2013-3618

[28] De Deken X, Wang D, Many MC, et al Cloning of two human thyroid cDNAs encoding new members of the NADPH oxidase family. J Biol Chem. 2000;275(30):23227‐23233. 10806195 10.1074/jbc.M000916200

[29] Dupuy C, Ohayon R, Valent A, Noel-Hudson MS, Deme D, Virion A. Purification of a novel flavoprotein involved in the thyroid NADPH oxidase. Cloning of the porcine and human cdnas. J Biol Chem. 1999;274(52):37265‐37269. 10601291 10.1074/jbc.274.52.37265

[30] Moreno JC, Pauws E, van Kampen AH, Jedlickova M, de Vijlder JJ, Ris-Stalpers C. Cloning of tissue-specific genes using serial analysis of gene expression and a novel computational subtraction approach. Genomics. 2001;75(1-3):70‐76. 11472069 10.1006/geno.2001.6586

[31] Revised guidelines for neonatal screening programmes for primary congenital hypothyroidism. Working group on neonatal screening of the European society for paediatric endocrinology. Horm Res. 1999;52(1):49‐52. 10640901 10.1159/000023433

[32] LaFranchi S . Congenital hypothyroidism: etiologies, diagnosis, and management. Thyroid. 1999;9(7):735‐740. 10447022 10.1089/thy.1999.9.735

[33] Peters C, Schoenmakers N. MECHANISMS IN ENDOCRINOLOGY: the pathophysiology of transient congenital hypothyroidism. Eur J Endocrinol. 2022;187(2):R1‐r16. 35588090 10.1530/EJE-21-1278 PMC9254299

[34] Carvalho DP, Dupuy C. Role of the NADPH oxidases DUOX and NOX4 in thyroid oxidative stress. Eur Thyroid J. 2013;2(3):160‐167. 24847449 10.1159/000354745 PMC4017742

[35] Weyemi U, Caillou B, Talbot M, et al Intracellular expression of reactive oxygen species-generating NADPH oxidase NOX4 in normal and cancer thyroid tissues. Endocr Relat Cancer. 2010;17(1):27‐37. 19779036 10.1677/ERC-09-0175

